# Hospital Needs and Hospital Appeals

**Published:** 1917-12-15

**Authors:** 


### HOSPITAL NEEDS AND HOSPITAL APPEALS.

### Hospitals with 150 Beds and Upwards.

**BROMPTON HOSPITAL FOR CONSUMPTION AND DISEASES OF THE CHEST.**—Brompton is the leading hospital for diseases of the chest, drawing its patients from all over the country. Eleven years ago it began to send its convalescent patients out to virgin forest at Frimley, Surrey, to work themselves back to health in clearing the waste and making it a productive garden. The twofold institution—hospital and working sanatorium—is the latest scientific method of restoring consumptive sufferers to health and a useful life. Years of success have proved it ideal. In the early days of the war the resources of the Brompton Hospital were placed at the disposal of the Admiralty and War Office authorities for the treatment not only of sailors and soldiers invalided with consumption, but also of their wives and families should treatment, which this hospital with its sanatorium at Frimley is in an unrivalled position to give, be needed. An additional annual income of £10,000 is now most urgently needed. Mr. Frederick Wood is the Secretary.

**GREAT NORTHERN CENTRAL HOSPITAL, HOLLOWAY ROAD, N.**—This institution serves the extensive and very poor districts of North London. The work is ever increasing, and proportionate growth has to be made to meet the demand. There are now special departments for the diseases of women, children, eye, ear, and throat, skin, and teeth; also for tuberculosis and venereal diseases. Up-to-date departments are provided for $x$-ray, massage, and electrical therapeutic treatment. Situated as it is in the best possible centre for its work, it is consequently unfavourably placed for coming before the eye of the wealthy and charitable public. The endowment is very small, and a yearly £35,000 of the expenditure has to be met by voluntary contributions. In addition to its work for the civilian population, 270 beds have been allotted for wounded soldiers. £5,000 is urgently needed before the close of the year, and gifts will be gratefully acknowledged if sent to Mr. Gilbert G. Panter, Secretary.

**GUY'S HOSPITAL, S.E. 1.**—In-patients to the number of 9,371 and over 106,000 out-patients were relieved by this famous institution in 1916 at a cost of £89,943, and with an assured income of less than £50,000. Owing to the increased cost of maintenance and a largely diminished income from legacies, the deficiency in the present year will be serious. The Governors will therefore greatly welcome any help at this season towards maintaining the full service of the hospital. Mr. H. Cosmo O. Bonsor is the President. Treasurer, Viscount Goschen. Bankers: The Bank of England.

**KING'S COLLEGE HOSPITAL, DENMARK HILL, S.E. 5,** is appealing for £60,000 to pay off the debt on the new buildings, and £15,000 extra annually to provide for current expenses, as its present assured income is very small. £5,000 is urgently required before the end of the year to enable the hospital to meet its immediate obligations. Donations should be forwarded to the Chairman, King's College Hospital, Denmark Hill, S.E. 5.

**LONDON HOMŒOPATHIC HOSPITAL.**—The board of management make a special appeal for aid in making good the large deficits incurred during the past few years. Eighty-five beds have been placed at the disposal of the Admiralty for sick and wounded sailors, of whom some 1,000 have already been treated to date. The receipt of contributions, addressed to the Earl of Donoughmore, the Treasurer, at the hospital, Great Ormond Street, W.C. 1, will be gratefully acknowledged.

**LONDON HOSPITAL, WHITECHAPEL, E.**—The needs of this institution are very much the same as they were last year, and very much the same as the needs now felt by most hospitals. Donations and subscriptions are almost as high as they have ever been, but the chief difficulty is the extraordinary rise in expenditure, due to the increased prices, and it would seem that the general increases in price of commodities touches chiefly those with which the hospital has to deal. It is more expensive to provide hot water and to warm the wards because of the price of coal; it is more expensive to feed the patients because milk and bread and fish and meat and vegetables have all become dearer. A new item of expenditure in hospital accounts is that which has to be paid in the way of salaries, and is directly due to the war, which has taken away much unpaid service. The hospital also has to meet expenditure in that it provides certain payments to wives and families of those of its staff who have been called to the Front. These are some of the reasons why the hospitals need very warm support at this time. House Governor, Mr. E. W. Morris.

**NATIONAL HOSPITAL FOR THE PARALYSED AND EPILEPTIC.**—The annual expenditure of this hospital has risen from £18,000 to close upon £30,000, mainly in consequence of the increase in the prices of food and drugs. In the large out-patient department (50,000 attendances are made annually) a considerable quantity of bromides is prescribed, and the rise in price of this indispensable article has had a material effect on the accounts. Seventy soldiers suffering from shell shock, blindness, mutism, deafness, nerve injuries, and general nervous complaints are to be found in the wards. The beds have been increased to 170 to meet the extra call for in-patient treatment. A sum of £10,000 is required to meet obligations to the bank and to tradesmen. The Secretary is Mr. Godfrey H. Hamilton, Queen Square, W.C.

**ROYAL FREE HOSPITAL, GRAY'S INN ROAD, W.C.**—The committee are making strenuous efforts to reduce the debt to the hospital bankers, at present amounting to £19,000, and they urgently need support for maintenance, and for extension of the maternity and infant welfare work. Mr. Reginald R. Garratt, Secretary.

**ST. BARTHOLOMEW'S HOSPITAL, WEST SMITHFIELD, E.C.**—This hospital is in need of much additional help. The maintenance expenditure has been increased by not less than £20,000 during the present year as the result of the abnormal advance in the cost of most articles of daily consumption, coupled with other unavoidable charges directly attributable to the war. Treasurer, the Viscount Sandhurst, P.C.; bankers, the Bank of England.

**ST. GEORGE'S HOSPITAL, HYDE PARK CORNER, S.W. 1.**—The Secretary appeals for cigarettes, tobacco, games, etc., for the wounded and sick sailors and soldiers who are being treated at the hospital. Contributions also are urgently required for the general maintenance of the institution, as there is a large overdraft at the bankers which has been caused principally by the enormous increase in the cost of all things necessary for the efficient treatment of the patients. The energetic help of all who have the interests of the hospital at heart is desired in order that its good work for the sick poor at home may not be impaired.

(*Continued on page* 237.)

**FURTHER HOSPITAL APPEALS—(*continued*).**

(*Continued from page* 226.)

**ST. MARY'S HOSPITAL, PADDINGTON, W.**—The regular sources of income yield about £18,000 only. A further sum of £17,000, therefore, is required every year. While this hospital is doing a great work for the war, its annual subscribers have fallen off through the war to the extent of more than £500 a year. Its war work consists not only in the devotion of one-third of its beds to the treatment of wounded soldiers from the Front, but in the manufacture and supply of anti-typhoid and other vaccines to all the Allied Armies in the field. Millions of doses have been supplied since the commencement of the war. To this work is due, in great measure, the striking rarity of typhoid fever in the Allied Forces. Secretary, Mr. Thomas Ryan.

**SEAMEN'S HOSPITAL, GREENWICH, S.E., AND ALBERT DOCKS.**—350 patients are under treatment in these hospitals. About half the number are Naval ratings from the Fleet, the other half being the equally gallant men of the Merchant Service, to whose courage and contempt of danger we all owe so much. Secretary, Mr. P. Michelli, C.M.G.

**WESTMINSTER HOSPITAL, BROAD SANCTUARY, S.W.**—Since the beginning of the year 383 military patients have been received, and owing to the great advance in the price of supplies an appeal is made for generous support. Secretary, Mr. S. M. Quennell.

**ROYAL HOSPITAL FOR INCURABLES, PUTNEY HEATH.**—£35,000 is needed annually to provide a home for incurables above the pauper class, and for those having a home but no means of support, a pension of £20 a year. Mr. Charles Cutting, Secretary. Offices, 4 St. Paul's Churchyard, E.C. 4.

### Hospitals under 150 Beds.

**THE CHELSEA HOSPITAL FOR WOMEN.**—This hospital has suffered especially through the effects of the war. To the difficulties of providing for current needs under the high prices that have prevailed has been added the necessity of obtaining contributions in reduction of the debt on the new building. This now amounts to £16,000, whilst the deficit on the maintenance account has reached the sum of £5,500. The work of this institution has, of late, been considerably increased by the reception of wounded officers into a number of its beds. The benefits the hospital confers on suffering womanhood have a particular bearing on the strength and welfare of the rising generation. There is a convalescent home at St. Leonards-on-Sea. Contributions to both institutions will be gratefully received by the Hon. Treasurer, Mr. Sidney H. Goldsmid, or the Secretary, Mr. H. H. Jennings, at the hospital, Arthur Street, Chelsea, S.W. 3.

**CITY OF LONDON LYING-IN HOSPITAL.**—The work which this hospital performs, in preserving the infant life of the community and caring for the mothers at the critical time of childbirth, is now, more than ever before, one of the most important problems of the day, and the nature of it must appeal strongly to the human side of everyone. There is now outstanding a loan of £7,000, most of which was incurred during the rebuilding of the hospital, which became imperative when the erection of a Tube station opposite caused serious damage to the foundations of the old building in 1907. It is also estimated that there will be a deficit of £1,500 at the end of the year. To maintain the service of the hospital at its present high state of efficiency it is absolutely essential that it receive greater support from the public. Secretary, Mr. E. Lionel Brown.

**EAST LONDON HOSPITAL FOR CHILDREN, SHADWELL, E. 1.**—This hospital stands in urgent need of help. In spite of the most economical management it is more than ever impossible to make both ends meet. Owing to the enormously enhanced prices of every commodity, this hospital, like all children's hospitals, has a special claim on the public to-day, for it is to the children that we look for our future armies and the future mothers of our race. The committee hopes that all kind friends will send an extra donation for next year, which is the Jubilee year of the hospital. All gifts will be gratefully received and acknowledged by the Secretary, Mr. W. M. Wilcox.

**HOSPITAL FOR EPILEPSY AND PARALYSIS.**—During the course of the last half-century the staff of the Hospital for Nervous Diseases, Maida Vale, have been acquiring that knowledge and skill which is enabling them to do so much for war wrecks both at Maida Vale and at the Golders Green branch. There are eighty-five beds at Maida Vale, including thirty-five for war wrecks from the trenches, and twenty-five for sailors, and 140 beds at Golders Green for discharged sailors and soldiers who need further treatment to fit them for return to civil life. Contributions towards the £8,000 requisite for carrying on the hospital's work are earnestly solicited. Mr. H. W. Burleigh is the Secretary.

**THE INFANTS' HOSPITAL, VINCENT SQUARE, WESTMINSTER, S.W. 1.**—The work of this hospital in saving infant life is now of greater importance than ever; but the hospital is entirely without funds for maintenance, and in debt to the extent of £3,500—a much larger sum than at any previous time. The Secretary is Mr. Alfred H. Small.

**MOUNT VERNON HOSPITAL FOR CONSUMPTION, NORTHWOOD, and FITZROY SQUARE, LONDON, W.,** is in great need of annual subscriptions and donations. Owing to the increased cost of maintenance, the expenditure is greatly in excess of the income for the current year. The treatment of women and children is a special feature. A large number of discharged soldiers are received, and more than 1,500 cases have been referred by the military authorities for special attention in the out-patient department during this year. Mr. W. J. Morton, Secretary.

**NATIONAL HOSPITAL FOR DISEASES OF THE HEART, WESTMORELAND STREET, W.**—This is the only institution of its kind in the United Kingdom, being the sole special hospital devoting its work to the alleviation of those suffering from diseases of the heart. The great increase in the price of everything required for the patients places the hospital in a position of much difficulty. New subscribers are very much wanted. About 10,000 recruits with doubtful hearts have been examined and reported on for the Army authorities. A ward, hitherto unused, has been given up for sailors and soldiers discharged on account of heart trouble.

**PASSMORE EDWARDS HOSPITAL, HARLESDEN ROAD, WILLESDEN, N.W.**—Owing to the rapidly increasing population of Willesden, which now numbers 160,000, the above hospital, the only general hospital in the district, finds itself hard pressed to deal with the many serious cases which are daily seeking admission, and which, owing to want of room, are unfortunately unable to be accommodated. The hospital, always open for inspection, has seventy-two beds, fifty-four of which are available for wounded soldiers. Donations for the build-

FURTHER HOSPITAL APPEALS—(*continued*).

ing or maintenance fund will be most gratefully received by the Secretary.

**POPLAR HOSPITAL FOR ACCIDENTS.**—The hospital issues a leaflet containing reasons for helping, amongst which is the fact that the institution is situated in the centre of the Port of London, and is in the best possible position for dealing with the serious accidents—owing to the war pressure, more numerous and serious than ever—which occur at the docks and at the hundreds of factories in the East End of London. One day alone twenty-one little children and several adults, victims of an aeroplane raid, were brought in—three were dead and five more died. The number of beds has been increased to 115, including twenty-four for wounded soldiers. Over 1,600 wounded men since December 1914 have been treated as in- and out-patients. The hospital has never been in debt, but though out of debt, it is not out of date. Funds for maintenance are asked for. Mr. Percy Rogers is the Secretary.

**QUEEN CHARLOTTE'S LYING-IN HOSPITAL, MARYLEBONE ROAD, N.W.**—The demands on the assistance of Queen Charlotte's Lying-in Hospital, Marylebone, during the present year have been larger than ever before, owing to the fact that the hospital is admitting a great number of the wives of our sailors and soldiers as well as Belgian and other refugees. Since the outbreak of war over 5,000 wives of our sailors and soldiers have either been received into the hospital or attended in their own homes. There is at present a debt of over £10,000 on the General Maintenance Account. The hospital has recently opened a new (temporary) building adjoining the hospital for the ante-natal department, where this most important work is now carried on under more favourable conditions than formerly. An Infant Consultation Centre has also been established. When so many lives are being laid down in the service of the country it is more than ever necessary to save the children, and Queen Charlotte's Hospital deserves the liberal support of the public to enable it to carry on satisfactorily its increasing and valuable work. Mr. Arthur Watts is Secretary.

**QUEEN'S HOSPITAL FOR CHILDREN, Bethnal Green, E. 2.**—An appeal has been issued by the Mayors of Bethnal Green, Shoreditch, Stoke Newington, and Hackney, appealing for funds to carry on the work of this, one of the largest institutions that minister to our little ones. Secretary, Mr. T. Glenton-Kerr.

**ROYAL HOSPITAL FOR DISEASES OF THE CHEST, CITY ROAD.**—Mr. Stuart de la Rue, the Chairman, urges that it would be nothing short of a calamity affecting the dignity of the nation if this hospital, under the patronage of our beloved Sovereign, should close for want of funds, and the more so should this happen after its splendid work for over a century, and at a time, too, when the need for its work is at the greatest. Every donation will now help to heal sick and wounded from the battlefields; it will also prepare the hospital for its equally great work after the war. At the request of the War Office thirty additional beds have been added.

**ST. JOHN'S HOSPITAL, LEWISHAM, S.E.**—At the outbreak of the war the number of beds was increased from forty-six to sixty, and later to sixty-two, thirty-eight of which were offered to the War Office and accepted free of charge, but later the committee were reluctantly compelled to apply for a grant; 111 wounded were, however, treated gratis during the first year, and cases continue to be received, thirty being at present in the hospital. For the above reasons the committee appeal to the charitably disposed to help this good work. Mr. F. W. Fryatt is the Hon. Secretary.

**ST. MARK'S HOSPITAL, CITY ROAD.**—The greatest need at present is the help of the charitable public in securing the necessary funds to continue our work in curing and relieving a large number of cancer and other rectal cases. The increased prices of all articles of food, etc., has naturally increased the expenditure, and as the war continues this difficulty will, of course, be greater. Therefore increased generous help is asked for. The Secretary is Mr. H. W. G. Coope.

**SAMARITAN FREE HOSPITAL FOR WOMEN, MARYLEBONE ROAD, N.W.**—The committee of management of the Samaritan Free Hospital for Women, the only entirely free hospital for women in London, appeal for Christmas benefactions to enable them to meet the outstanding tradesmen's accounts amounting to £1,400 and to reduce the bankers' loan of £500. Special contributions are earnestly solicited towards the endowment of a ward for cancer, of patients suffering from which disease upwards of 100 cases were treated last year, and many are waiting admission. Two beds have been named, and an earnest appeal is made for special donations in order that the ward may be opened.

**SOUTH LONDON HOSPITAL FOR WOMEN.**—This new hospital, opened by the Queen in 1916, has eighty beds for women and children, and is entirely officered by medical women. A special feature is the provision of sixteen beds in private wards for women of limited means at low inclusive fees. The increasing numbers of women and children attending the out-patient department at 88-90 Newington Causeway have necessitated an extension of the premises by renting an adjoining house —86 Newington Causeway. This extension, while enabling the work to be carried on under less crowded conditions, in no way lessens the need for an entirely new outpatient department. The board of management earnestly appeal for contributions and promises of help, which will be gladly received by the Secretary of the Hospital, South Side, Clapham Common, S.W.

**VICTORIA HOSPITAL FOR CHILDREN, CHELSEA, S.W.**—The committee find themselves at the end of their financial resources, and will have to obtain a loan from their bankers to meet the current expenses, unless some unforeseen help is received. The year 1917 began with a deficit of £448, which will be very much larger by December 31. The increase in the cost of all commodities has added enormously to the cost of maintenance, and the present income is now wholly insufficient to meet the expenditure. At least £1,000 is required to meet the immediate needs of the hospital. Mr. H. G. Evered is the Secretary.

### Hospitals under 50 Beds.

**THE BELGRAVE HOSPITAL FOR CHILDREN, CLAPHAM ROAD, S.W.**—This hospital's work has been carried on undiminished; and—thanks to generous friends —its income has been well maintained; but, owing to the high prices of food, drugs, and in fact all necessities, there is already a deficiency of £1,500 on this year's working. Mr. Alfred H. Small is the Acting Secretary.

**GROSVENOR HOSPITAL FOR WOMEN.**—The immediate requirement of this institution is money. The debt to the bank is now about £250, although an unexpected

